# Ocular Adverse Effects in Atopic Dermatitis Patients Treated With Dupilumab: A Bibliometric Analysis

**DOI:** 10.3389/fmed.2022.802036

**Published:** 2022-03-03

**Authors:** Qian-Nan Jia, Ju Qiao, Kai Fang, Yue-Ping Zeng

**Affiliations:** Department of Dermatology, State Key Laboratory of Complex Severe and Rare Diseases, Peking Union Medical College Hospital, Chinese Academy of Medical Science and Peking Union Medical College, National Clinical Research Center for Dermatologic and Immunologic Diseases, Beijing, China

**Keywords:** atopic dermatitis, dupilumab, ocular adverse effects, bibliometric, conjunctivitis

## Abstract

**Background:**

Atopic dermatitis (AD) is one of the most common chronic inflammatory skin disorders. Dupilumab, the first targeted biological drug approved for the treatment of AD, has been widely used, along with increasing ocular adverse effects (AEs).

**Objective:**

To perform a bibliometric analysis of all the qualified literature involving ocular AEs during the treatment of AD with dupilumab.

**Methods:**

Relevant studies were extracted from the Web of Science database and screened by researchers. The bibliographic analysis was performed using the VOSviewer.

**Results:**

A total of 138 articles were enrolled in this study. The first study was published in 2016 by Oregon Health and Science University from the United States. The majority of publications were published in the past 3 years. *British Journal of Dermatology* published the highest number of articles. The United States was the country with the most publications. Sanofi (France) and Regeneron Pharmaceuticals (USA) were the leading organizations with the most contributions. Conjunctivitis was the most common ocular AE. The management of AD will continue to be the research hotspot and development trend in this area. The milestone research is the first article “Two Phase 3 Trials of Dupilumab vs. Placebo in Atopic Dermatitis” published in the *New England Journal of Medicine*. Most of the top 10 papers were mainly randomized, placebo-controlled phase 2 and phase 3 clinical trials and real-life large cohort studies.

**Conclusions:**

This study may help better understand ocular AEs in the dupilumab treatment of AD, and grasp the research trends and most influential topics in this field.

## Introduction

Atopic dermatitis (AD) is one of the most common chronic inflammatory skin disorders, and affects patients of all ages in all aspects, from physical health to psychological condition and economic burden (1, 2). The prevalence of AD is up to 25% in children (3) and 7–10% in adults (4). Most patients have an early disease onset by the age of 5 years and may last for a lifetime. Clinically, AD is characterized by erythema and severe pruritus, and had a strong tendency to relapse. Patients with AD suffer from intense itching, pain, sleep disturbance, anxiety, depression, and psychosocial stress (5, 6). With increased prevalence for years, AD has been becoming a health-threatening disease with severe impacts on the patient's quality of life.

Atopic dermatitis is believed to be the result of complex interactions between genetic and environmental factors that affect the immune system and epidermal barrier function (7). The inflammatory reaction in AD is generally considered due to the activation of T helper cell type 2 (Th2) immune response, in which related cytokines interleukin-4 (IL-4), IL-13, and IL-31 are quite essential (8). The treatment of AD remains to be a clinical challenge, especially for patients with moderate-to-severe AD. Moderate-to-severe AD may require systemic agents, such as immunosuppressant drugs and dupilumab (9). Dupilumab is a fully-human monoclonal antibody that is against IL-4 receptor α and blocks crucial pathways from both IL-4 and IL-13 in AD (10). The safety profile and adverse effects (AEs) are required in the long-term dupilumab treatment of patients with AD.

In 2014, Beck et al. (11) first reported that patients with moderate-to-severe AD had marked and rapid improvement after the treatment with dupilumab in a randomized, double-blind, placebo-controlled trial. Moreover, 2 years later, two-phase 3 trials of dupilumab vs. placebo in AD investigated the effectiveness and safety of dupilumab, and revealed that dupilumab improved the signs and symptoms of AD in all aspects (12). Since then, articles involving the safety of patients with AD treated with dupilumab have been published increasingly. With the approval of dupilumab for the treatment of moderate-to-severe AD adults by the FDA in 2017, dupilumab has been used widespread, along with increasing AEs reported in the literature. AEs induced by dupilumab in AD clinical trials were mainly ocular diseases and increased eosinophil counts. Other AEs reported later in the real world were as follows: psoriasis-like lesions, head and neck erythematous lesions, rosacea-like skin symptoms, alopecia, muscular pain, and arthritis (13–19). In detail, ocular AEs mainly included conjunctivitis, keratitis, keratoconjunctivitis, blepharitis, eye pruritus, and dry eye. The prominent clinical manifestation of ocular AEs was redness of the conjunctiva in both eyes, and especially hyperemia and nodular swelling of the limbus. Other clinical symptoms included itching, tearing, stinging, burning, and foreign body sensation. Bibliometrics is a quantitative analysis of the published literature in a specific scientific field using mathematical and statistical methods. The application of bibliometric analysis could help better understand the knowledge structure and significant advances in a certain research field, and is thus rapidly increasing in several diseases for the past few years. However, there are few bibliometric studies referring to AD. Therefore, we aimed to perform a bibliometric analysis of all the qualified literature involving ocular AEs during the treatment of AD with dupilumab. The present study described the characteristics of articles and the collaboration network of authors, organizations, and countries/regions, and revealed the research dynamics, especially evaluating research focus and emerging trends in this field.

## Methods

### Data Extraction and Screening

Relevant studies in relation to dupilumab in the treatment of atopic dermatitis were extracted from the Web of Science Core Collection database on August 30, 2021. The detailed search strategy was as follows: TOPIC: (dupilumab) *AND* TOPIC: (atopic dermatitis) *AND* DOCUMENT TYPES: (Article OR Letter) *AND* LANGUAGE: (English).

Two researchers (QN Jia and J Qiao) further performed literature screening of the title and abstract independently, with full text downloaded if necessary. Research regarding ocular adverse events in the treatment of atopic dermatitis with dupilumab were retrieved and collected, such as research letters and case letters. Articles that meet the following criteria were excluded: (1) ocular adverse events were uninvolved in the main topic of article; (2) the article was a review, systematic review, meta-analysis, correspondence, expert opinion, retracted article, conference article, or guideline. With the guidance of a senior expert (YP Zeng), a final agreement was reached on the literature screening.

For articles that met the criteria, we recorded all the available information, such as title, authors, institutions, funding organization, country/region, abstract, keywords, journal of publication, year of publication, citations, and cited references. Journal impact factor (IF) was queried from the 2020 Journal Citation Reports.

### Data Analysis and Visualization

The bibliographic information was analyzed using the VOSviewer (version 1.6.17). The co-occurrence analyses of authors, organizations, countries/regions, and keywords were performed with VOSviewer, as well as the analyses of reference citations, and co-citations. The related maps of the above analyses were produced.

## Results

**Figure 1:**
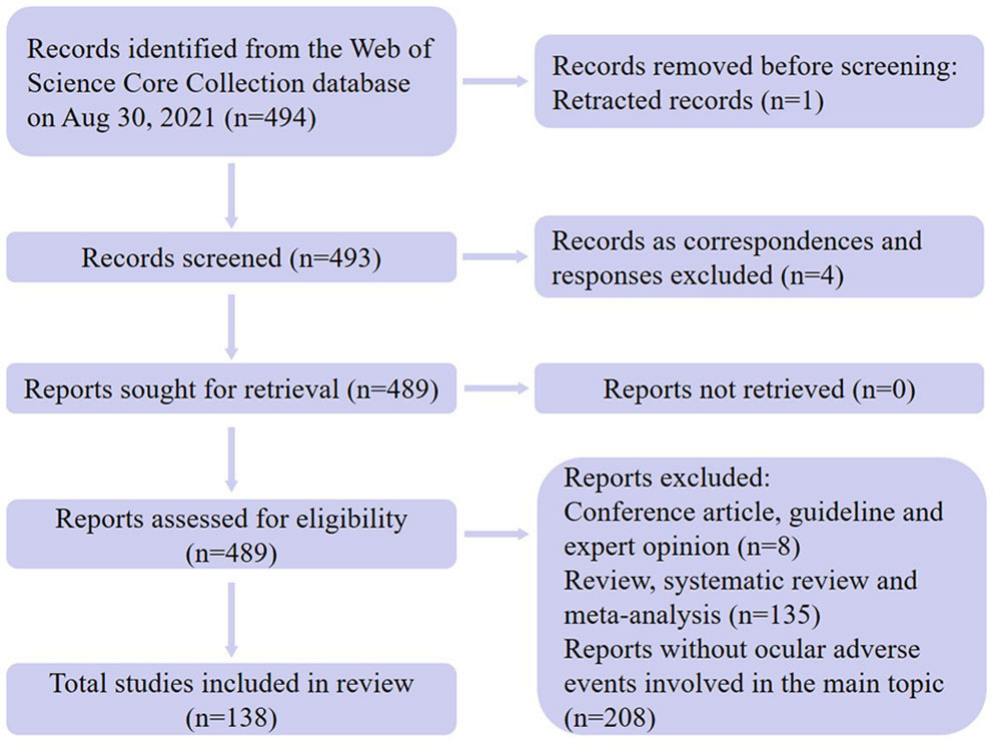
The flow diagram of this study.

We extracted 494 articles on the topic of patients with AD treated with dupilumab from the Web of Science Core Collection database following our search strategy. After literature screening by researchers, a total of 138 articles reported the occurrence of ocular AEs and were enrolled in this study. By document type, 138 articles were categorized into 97 original articles and 41 letters. The flow diagram of the present study is shown in Figure 1.

### Evolution of Scientific Production

**Figure 2:**
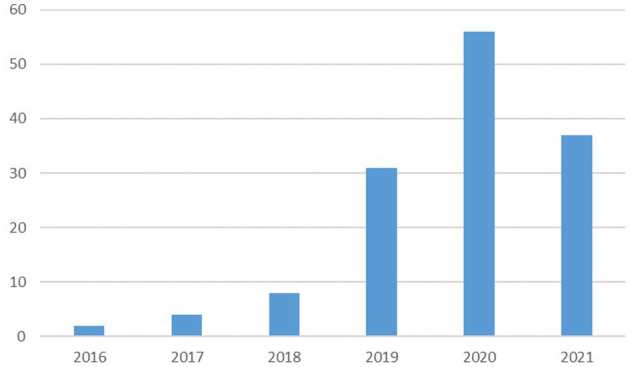
The number of publications of each year in this area.

All 138 articles were published between 2016 and 2021. The number of publications of each year are displayed in Figure 2. There were a few articles sporadically published from 2016 to 2018. However, the number of publications abruptly rose in 2019 and 2020, accounting for nearly two-thirds of the total amount, when added together. It should be noted that data extraction was performed in August 30, 2021, and thus publications in 2021 were incomplete.

### Journals of Publication

All the articles analyzed in this study were published in 47 journals, including 22 Dermatological journals. The other 25 journals were classified into Ophthalmology (9 journals), Medicine, General & Internal (6 journals), Immunology (6 journals), Allergy (5 journals), Pharmacology & Pharmacy (2 journals), Medicine, Research & Experimental (1 journal), and Multidisciplinary Sciences (1 journal) (Five journals were classified into both Allergy and Immunology categories.). The number of publications varied from 1 to 18 in these journals. The journal IF was distributed between 0.22 and 91.245. Table 1 showed that top 10 journals with the largest number of published articles, comprising nearly two-thirds of the total publications. Of the 10 journals, four journals had smaller IF than 5, three journals with an IF between 5 and 10, while three journals (*Allergy, Journal of the American Academy of Dermatology* and *JAMA Dermatology*) had an IF higher than 10. Eight out of the 10 journals belonged to a dermatologic field (80%). *British Journal of Dermatology* took the lead and had the highest number of publications (18, 13.04%, IF 2020 = 9.302), followed by *Journal of the American Academy of Dermatology* (15, 10.87%, IF 2020 = 11.527), *Dermatologic Therapy* (13, 9.42%, IF 2020 = 2.851), *Journal of the European Academy of Dermatology and Venereology* (11, 7.97%, IF 2020 =6.166), and *JAMA Dermatology* (7, 5.07%, IF 2020 = 10.282).

**Table 1:** The top 10 journals with the largest number of published articles in the study area.

**Journal**	**Publication number**	Journal IF^*^	**WoS^#^ Categories**
Br J Dermatol	18	9.302	DERMATOLOGY
J Am Acad Dermatol	15	11.527	DERMATOLOGY
Dermatol Ther	13	2.851	DERMATOLOGY
J Eur Acad Dermatol Venereol	11	6.166	DERMATOLOGY
JAMA Dermatol	7	10.282	DERMATOLOGY
Am J Clin Dermatol	5	7.403	DERMATOLOGY
Int J Dermatol	5	2.736	DERMATOLOGY
J Dermatol Treat	5	3.359	DERMATOLOGY
Allergy	4	13.146	ALLERGY, IMMUNOLOGY
J Clin Med	4	4.241	MEDICINE, GENERAL & INTERNAL

* *Journal impact factor (IF) was queried from the 2020 Journal Citation Reports*.

# *Web of Science*.

### Analysis of Countries/Regions, Organizations, and Authors

**Figure 3:**
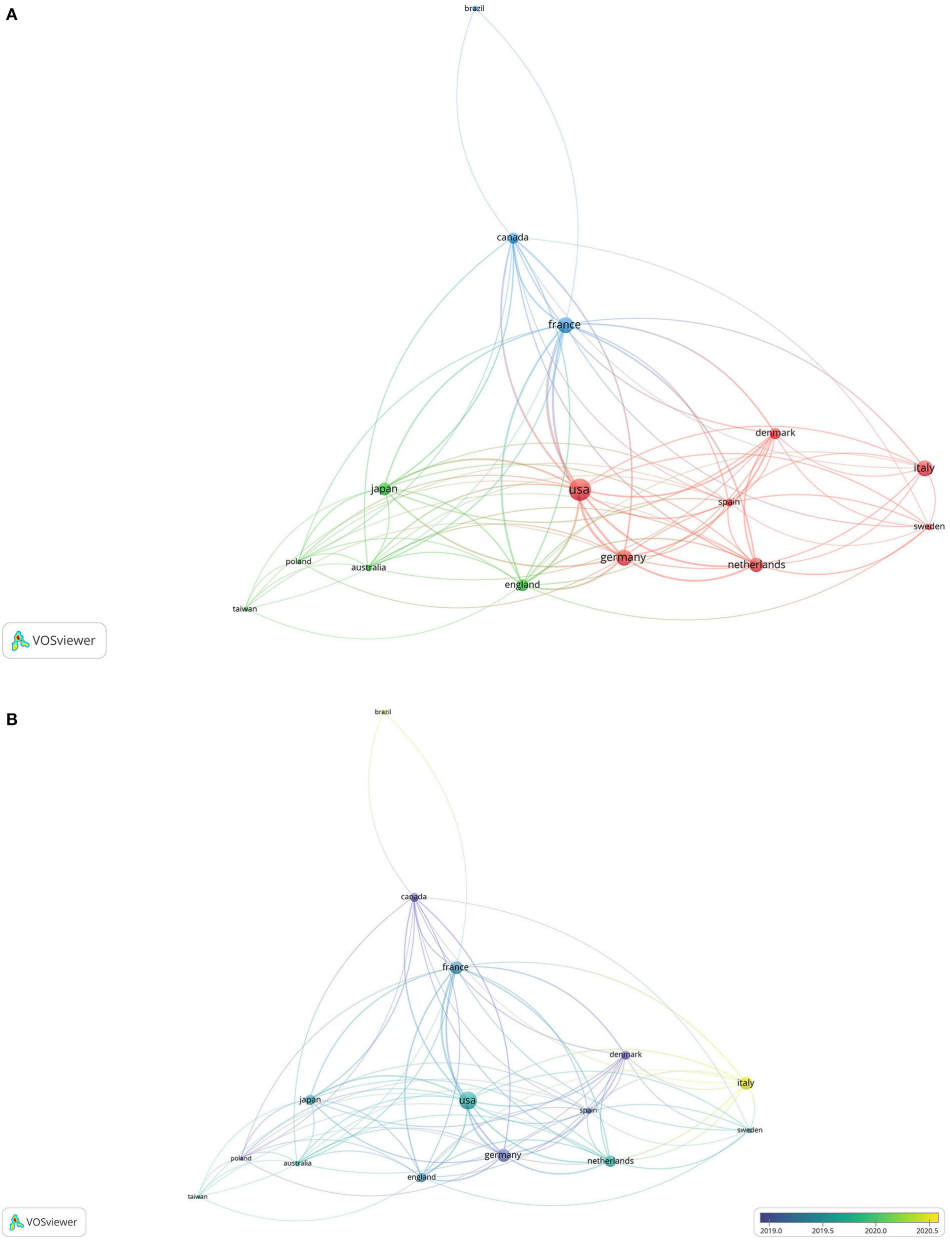
The collaborative network among the major countries/regions. **(A)** Network visualization; **(B)** overlay visualization.

There were 33 countries/regions reporting ocular AEs during the treatment of AD with dupilumab. The United States published the highest number of publications (53, 38.41%), followed by France (27, 19.57%), Germany (27, 19.57%), Italy (27, 19.57%), and Netherlands (22, 15.94%). The collaborative network among the major countries/regions was generated by VOSviewer and presented in Figure 3. There were 15 nodes and 80 links. The larger the circle, the more publications the country/region. A line is corresponding to a connection between two countries/regions. The length of a line represented the cooperation intensity of two countries/regions. The shorter the line, the stronger the relatedness. As shown in Figure 3B, the geographical distribution changed over time. The foremost contributing countries were the United States, Germany, Denmark, and Canada, followed by France, UK, Netherlands, Japan, etc. Italy subsequently caught up in 2020 with 27 publications.

**Figure 4:**
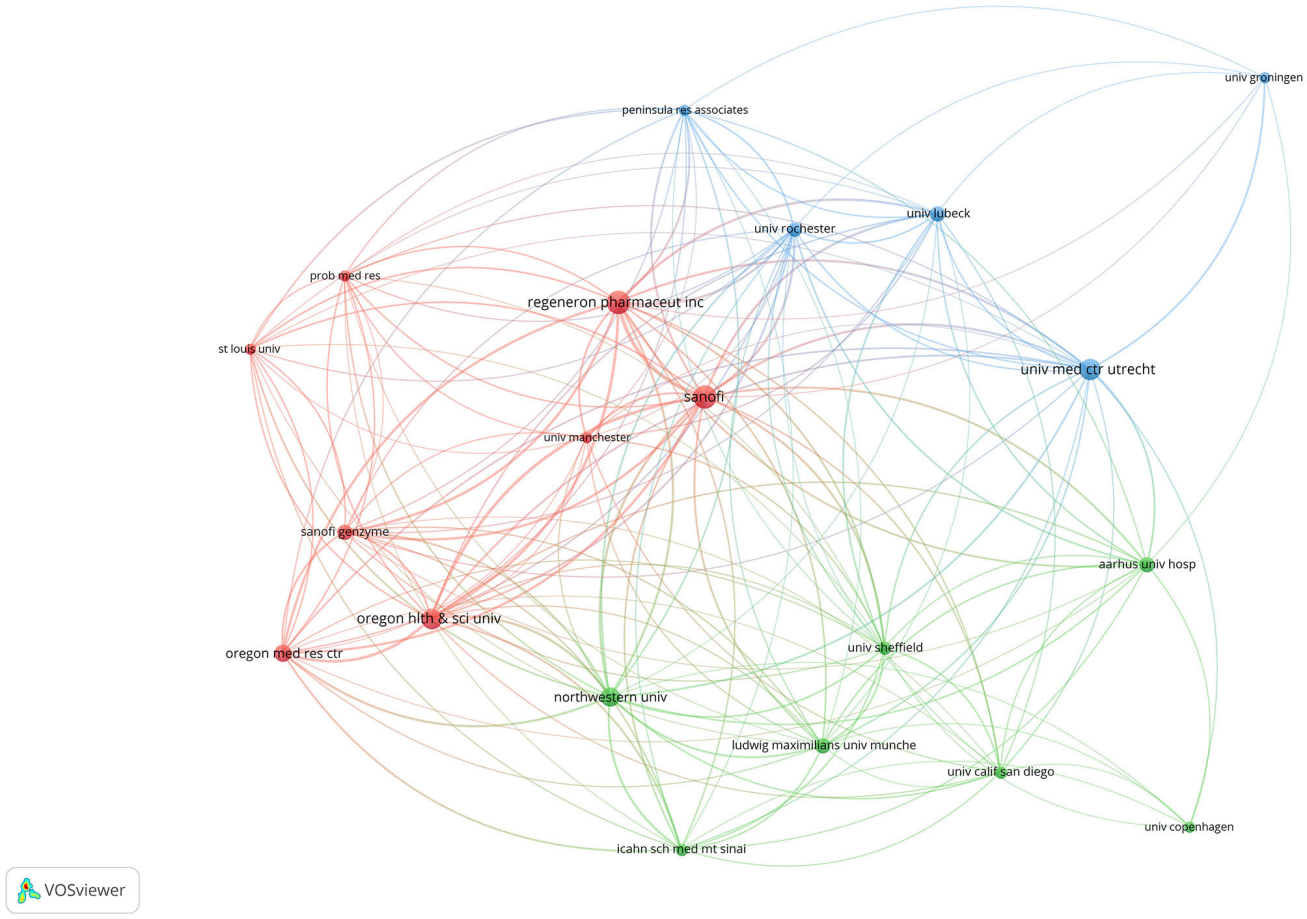
The collaborative network of organizations.

In total, 326 organizations had made contributions in this study area. Regeneron Pharmaceuticals Inc. made the most contributions with 21 articles (15.22%), followed by Sanofi (20, 14.49%), University Medical Center Utrecht (17, 12.32%), Oregon Health and Science University (16, 11.59%), and Northwestern University (14, 10.14%). Oregon Health and Science University was the first to report ocular AEs in this medication field, subsequently with Oregon Medical Research Center, Sanofi, Ludwig-Maximilians-University Munchen, and Aarhus University Hospital, etc. When it comes to the cooperation network, there were 20 nodes and 158 links (Figure 4). The organizations presented in Figure 4 were mainly from the United States and Italy, while others were from the UK, Denmark, and the Netherlands.

**Figure 5:**
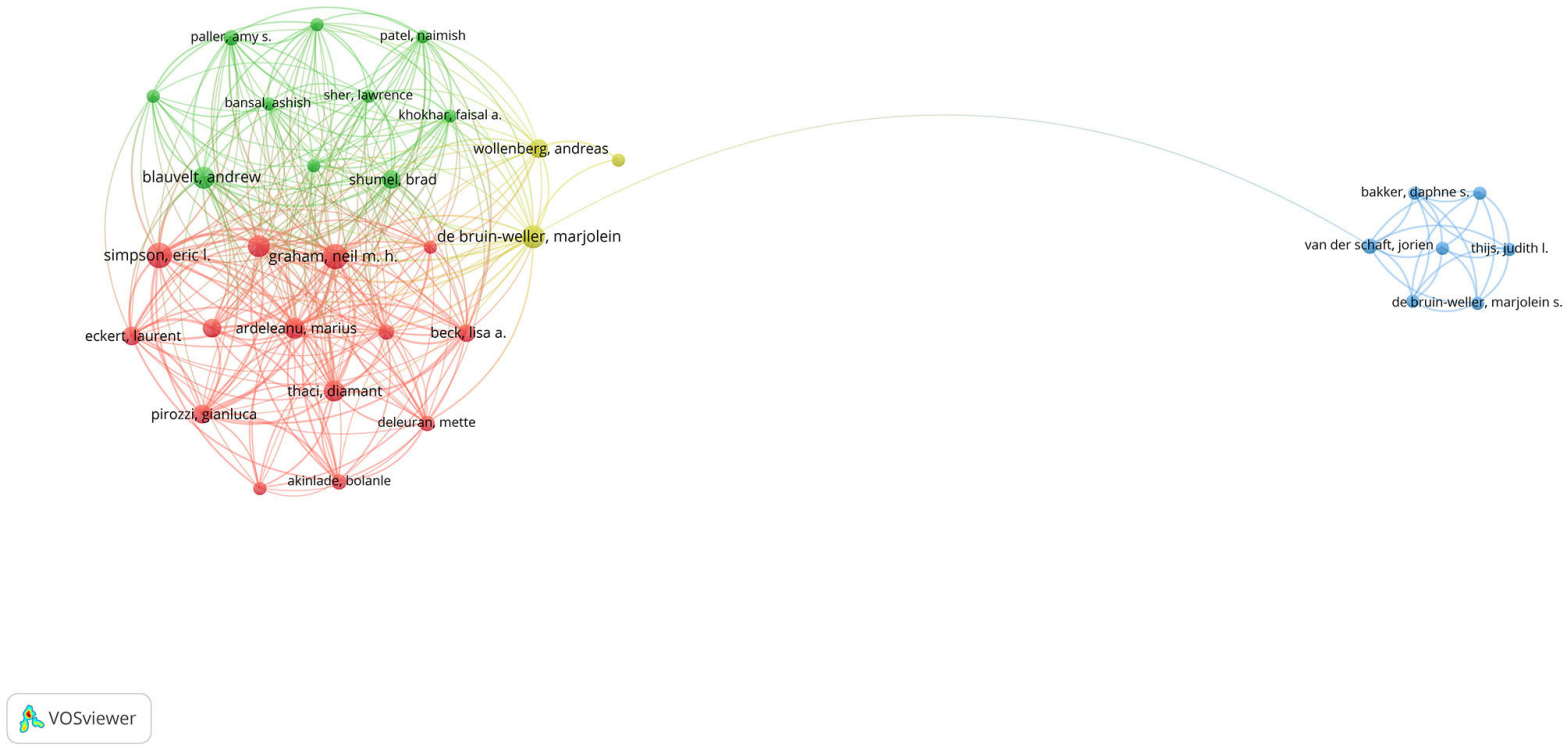
The collaborative network of authors.

A total of 788 authors participated in this research field. Both Graham NMH (Regeneron Pharmaceuticals Inc., USA) and Simpson EL (Oregon Health and Science University, USA) published the largest number of articles in this study area (10, 7.25%), followed by de Bruin-Weller M (9, 6.52%, University Medical Center, Netherlands), Blauvelt A (8, 5.80%, Oregon Medical Research Center Utrecht, USA), and Chen Z (8, 5.80%, Regeneron Pharmaceuticals Inc., USA). Co-authorship analysis revealed 34 nodes and 300 links (Figure 5). The top 10 authors with the most publications were mainly from the United States, with only three authors from the Netherlands, Germany, and France, respectively.

### Co-occurrence Analysis of Keywords

**Figure 6:**
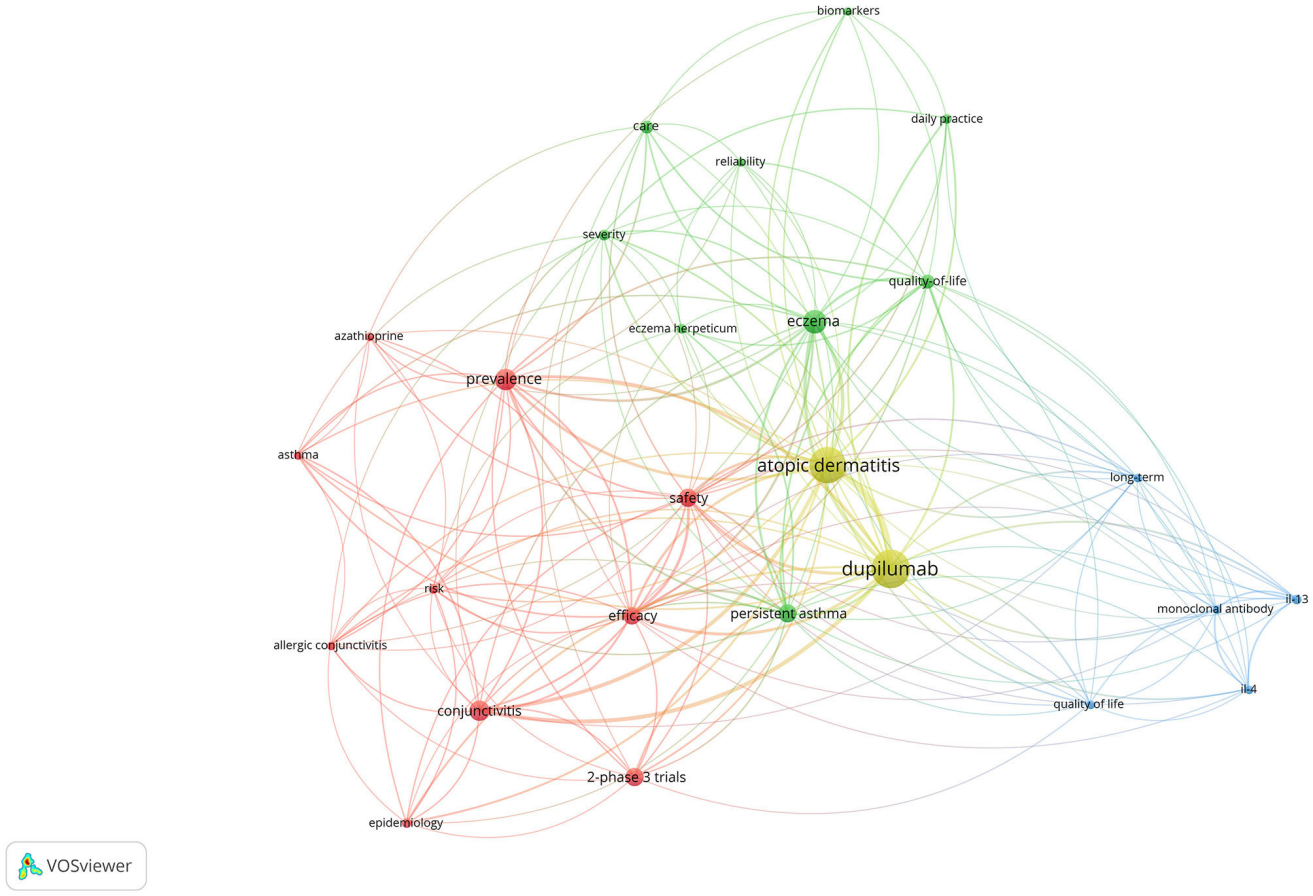
The co-occurrence network of keywords.

As shown in Figure 6, the co-occurrence analysis of keywords was conducted and revealed 26 keywords and 182 links. The top keywords with the highest frequency were as follows: prevalence, conjunctivitis, 2-phase 3 trials, persistent asthma, safety, efficacy, quality of life, care, severity, daily practice, eczema herpeticum, epidemiology, IL-13, risk, allergic conjunctivitis, asthma, azathioprine, biomarkers, IL-4, long-term, monoclonal antibody, and reliability. Conjunctivitis was the main ocular AE detected in this analysis. Prevalence is the most popular phrase, besides AD and dupilumab. Two-Phase 3 Trials of Dupilumab vs. Placebo in Atopic Dermatitis published in the New England Journal of Medicine were the most frequently mentioned clinical trials. Keywords regarding the management of AD made up the largest proportion, such as the safety and efficacy of dupilumab, quality of life and care of the patients, and daily practice. The mechanism of dupilumab in the treatment of AD was also commonly mentioned with the following keywords: IL-13, IL-4, biomarker, and monoclonal antibody.

### Reference Citations and Co-Citations

**Figure 7:**
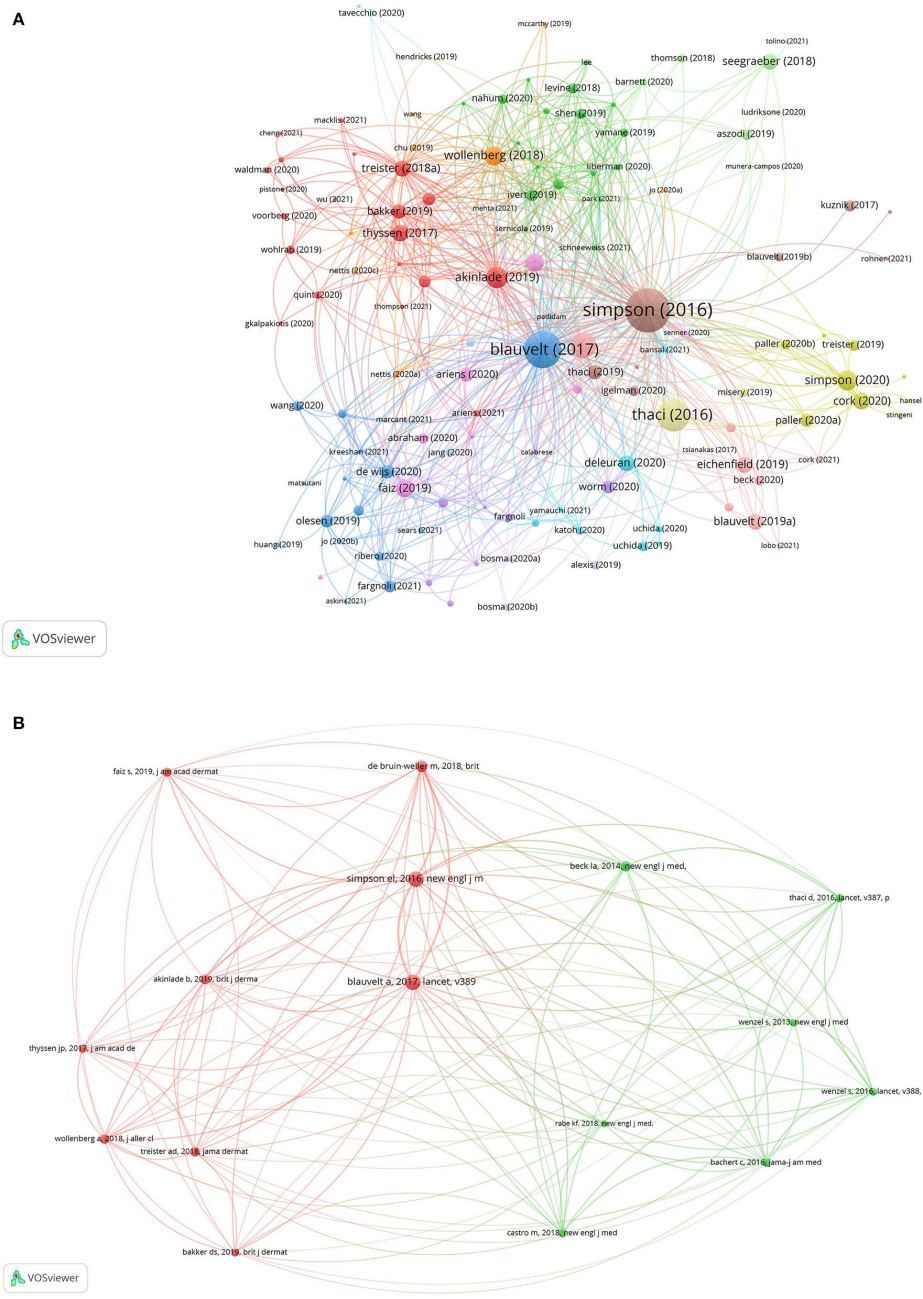
The analysis of reference citations **(A)** and co-citations **(B)**.

Of the 138 papers, the analysis of reference citations discovered 134 nodes and 686 links (Figure 7A). The vast majority of studies were published in 2019, 2020, and 2021, with only a few in 2016, 2017, 2018. The top 10 papers with most citations were uniformly distributed between 2016 and 2020. “Two-Phase 3 Trials of Dupilumab vs. Placebo in Atopic Dermatitis” (12) (2016), which was also detected as a popular keyword, had 741 citations. Other top papers were as follows: “Long-term management of moderate-to-severe atopic dermatitis with dupilumab and concomitant topical corticosteroids (LIBERTY AD CHRONOS): a 1-year, randomized, double-blinded, placebo-controlled, phase 2 trial” (20) (2017, 443 citations); “Efficacy and safety of dupilumab in adults with moderate-to-severe atopic dermatitis inadequately controlled by topical treatments: a randomized, placebo-controlled, dose-ranging phase 2b trial” (9) (2016, 315 citations). The above papers were published in the *New England Journal of Medicine* and *Lancet* with an IF of 91.245 and 79.321.

The co-citation analysis is performed with 20 citations set as the minimum number of a cited reference. Of the 1,348 cited references, 16 papers met the threshold with 120 links (Figure 7B, Table 2). All the papers were published between 2013 and 2019, with the majority between 2016 and 2019. More than half of these papers were published in the *New England Journal of Medicine* (5, 31.25%), *Lancet* (3, 18.75%), and *British Journal of Dermatology* (3, 18.75%). Other journals were the *Journal of the American Academy of Dermatology, Journal of Allergy and Clinical Immunology-In Practice, JAMA Dermatology*, and *JAMA*. The top 2 papers were identical to those in the analysis of citations, and were undoubtedly the most co-cited papers with 87 and 82 citations, respectively. “Dupilumab with concomitant topical corticosteroid treatment in adults with atopic dermatitis with an inadequate response or intolerance to ciclosporin A or when this treatment is medically inadvisable: a placebo-controlled, randomized phase III clinical trial” (21) (2018) ranked third with 49 citations; followed by “Dupilumab treatment in adults with moderate-to-severe atopic dermatitis” (11) (2014, 39 citations), and “Conjunctivitis in dupilumab clinical trials” (19) (2019, 35 citations).

**Table 2:** The top 5 articles in the analysis of reference co-citations.

**Year**	**Journal**	**References**	**Article title**	**Citations**	**Total link strength**
2016	New Engl J Med	Simpson et al. (12)	Two Phase 3 Trials of Dupilumab versus Placebo in Atopic Dermatitis	87	385
2017	Lancet	Blauvelt et al. (20)	Long-term management of moderate-to-severe atopic dermatitis with dupilumab and concomitant topical corticosteroids (LIBERTY AD CHRONOS): a 1-year, randomized, double-blinded, placebo-controlled, phase 2 trial	82	367
2018	Brit J Dermatol	de Bruin-Weller et al. (21)	Dupilumab with concomitant topical corticosteroid treatment in adults with atopic dermatitis with an inadequate response or intolerance to ciclosporin A or when this treatment is medically inadvisable: a placebo-controlled, randomized phase III clinical trial	49	252
2014	New Engl J Med	Beck et al. (11)	Dupilumab treatment in adults with moderate-to-severe atopic dermatitis	39	230
2019	Brit J Dermatol	Akinlade et al. (19)	Conjunctivitis in dupilumab clinical trials	35	169

## Discussion

Dupilumab which is the first targeted biological drug approved for the treatment of AD, has been generally administrated in both adults and children. Ocular AEs reported during the treatment of AD with dupilumab have been increasing sharply, and were proposed to be under the definition “dupilumab induced ocular surface disease” (DIOSD) by Zirwas et al. (22).

In recent years, bibliometric analysis has been gradually applied in a variety of medical fields, such as respiratory medicine, ocular disease, and cancer. In dermatology, several research fields have been analyzed with bibliometric methods, such as general dermatology, melanoma, psoriasis, psoriatic arthritis, toxic epidermal necrolysis, and Stevens-Johnson syndrome (23–25).

To the best of our knowledge, this is the first bibliometric analysis focusing on ocular AEs during the treatment of AD with dupilumab. In this study, we investigated published documents involving dupilumab in the treatment of AD, identified all the related ocular AEs, and systematically analyzed the basic characteristics of literature, such as the journals, collaborative networks of countries/regions, organizations and authors, co-occurrence of keywords, and citation network.

The analysis of document type showed that less than one-third of documents were case letters or research letters, and most papers were original research studies. With regard to the publication time, the earliest study was published in 2016, and the majority (89.86%) of publications were published in the past 3 years (2019–2021), indicating research on ocular AEs is a novel and emerging field.

The bibliometric analysis of journals found a total of 47 journals of publication. Dermatologic journals (22, 46.81%) and ophthalmologic journals (9, 19.15%) were undoubtedly the top 2 categories, followed by Medicine, General & Internal and Immunology/Allergy categories. The reason why Medicine, General & Internal journals published quite a few papers might be that some general medicine journals (*New England Journal of Medicine and Lancet*) have higher IFs than dermatologic journals. The top 6 journals were the *British Journal of Dermatology, Journal of the American Academy of Dermatology, Dermatologic Therapy, Journal of the European Academy of Dermatology and Venereology, JAMA Dermatology*, and *American Journal of Clinical Dermatology*. All of them were dermatologic journals, and 5 out of 6 journals were world-class dermatologic journals with IFs higher than 6.

Our study showed that 33 countries/regions had reported ocular AEs during the treatment of AD with dupilumab. The United States published the earliest article and the highest number of publications and was far ahead of other countries/regions. In the collaborative network, the United States had the strongest total link strength, suggesting a highest cooperation intensity with others. Although Italy tied for second with France and Germany in the publication number, Italy ranked 13th in the total link strength, which might be because studies from Italy were mainly published after 2020 with limited time for citations. These countries/regions presented in the collaboration network were mainly distributed in Europe (9/15), followed by North America (2), East Asia (2), Australia (1), and South America (1). The vast majority (86.67%) of countries/regions were in the Northern Hemisphere. Except for Brazil, all the other countries/regions were developed countries/regions.

In the bibliometric analysis of organizations, 326 organizations had made contributions in this study area. Oregon Health and Science University published the first article reporting ocular AEs during the treatment of AD with dupilumab. Most organizations were universities and research centers. However, organizations leading the way were Sanofi (France) and Regeneron Pharmaceuticals Inc. (USA), since dupilumab was jointly developed by them. University Medical Center Utrecht (Netherlands) was the leading university, followed by Oregon Health and Science University (USA), and Northwestern University (USA). Thus, the main forms of research group were biopharmaceutical companies, universities, and research centers. Regarding the cooperation network, Sanofi containing Sanofi Genzyme ranked first in the cooperation intensity with the highest total link strength, followed by Regeneron Pharmaceuticals Inc., demonstrating the dominant position in the development of dupilumab. Half of the top 10 organizations with the most citations were from the United States, reflecting the great influence on this research field.

On the subject of authors, we summarized a total of 788 authors participating in this research field. Concerning first authors, Graham NMH (Regeneron Pharmaceuticals Inc.) and Simpson EL (Oregon Health and Science University) were the most prolific authors with the most citations. In the co-authorship network, the top 10 authors with the highest link strength mainly came from organizations of the United States, in which Regeneron Pharmaceuticals Inc. made up the largest proportion. Additionally, the contributions of ophthalmology specialists are significant in the correct classification and management of eye involvement in the treatment of AD, and several articles have highlighted the significance of the cooperation between dermatologists and ophthalmologists (26).

With respect to the co-occurrence analysis of keywords, conjunctivitis was the most common ocular AE identified in this study. Plenty of clinical trials have demonstrated that AD patients treated with dupilumab had a higher incidence of conjunctivitis than those treated with placebo. The term “conjunctivitis” contained the following diseases: conjunctivitis, conjunctivitis allergic, conjunctivitis bacterial, conjunctivitis viral, and atopic keratoconjunctivitis, with all being reported as AEs in the literature. Other ocular AEs, such as dry eyes, eye pruritus, blepharitis, blepharoconjunctivitis, and cicatricial ectropion, were also reported.

Notably, in our study, allergic conjunctivitis, blepharitis, keratitis, and atopic keratoconjunctivitis were also complications at baseline in some cases. Previous studies have proved that the above ocular diseases are common comorbidities occurring in nearly half of patients with AD (27). Moreover, Akinlade et al. (19) has found that prior conjunctivitis history is associated with the increased incidence of conjunctivitis in AD patients treated with dupilumab. The mechanism of ocular AEs remains unknown. The possible causes of ocular AEs may be the inhibition of goblet cells through blocking IL-4 and IL-13 with dupilumab, resulting in decreased mucin secretion and mucosal epithelial barrier dysfunction (28). Other studies have found that the increased risk of conjunctivitis may be associated with serum IgE, thymus and activation-regulated chemokine in dupilumab treated patients (29). It is worth exploratory whether conjunctivitis results from the blockade of IL-4 and IL-13 with dupilumab or that conjunctivitis, in the first place, is part of this systemic allergic disorder.

Furthermore, keywords regarding the management of AD made up the largest proportion in the co-occurrence network of keywords, such as the safety and efficacy of dupilumab, quality of life and care of the patients, and daily practice. Clinical lesions of AD usually tend to be recurrent and may last for a lifetime. Moreover, topical treatments have relatively limited efficiency for moderate-to-severe AD, and systemic therapy with long-term significant efficiency and safety is required for treatment. Therefore, for a long time, the management of AD is one of the great concerns in dermatology, and remains to be a therapeutic challenge for clinicians. The occurrence network of keywords indicated that the management of AD will continue to be the research hotspot and development trend in this medical area.

Reference citation links are undirected links between two articles where one cites the other. The article “Two Phase 3 Trials of Dupilumab vs. Placebo in Atopic Dermatitis” published in the top-tier journal *New England Journal of Medicine*, took the lead with 741 citations, such as both citing and cited conditions. This article was the first study discovering ocular AEs of dupilumab in the treatment of AD, and thus became the milestone research in this field. The top papers with the most citations were mainly randomized, placebo-controlled phase 2, and phase 3 clinical trials. Except for clinical trials, real-life evidence and clinical reports have also provided fundamental evidence for future studies. A clinical report “Conjunctivitis occurring in atopic dermatitis patients treated with dupilumab-clinical characteristics and treatment” (30), ranked the seventh with 75 citations. This article reported conjunctivitis occurring in 25 and 50% of dupilumab-treated patients with AD in two centers from 2016 to 2017, with a focus on the detailed clinical characteristics. More importantly, the authors reported successful treatment of all the 13 patients with fluorometholone or tacrolimus, and gave dermatologists and ophthalmologists useful and fundamental recommendations. A large retrospective cohort study “Effectiveness and safety of dupilumab for the treatment of atopic dermatitis in a real-life French multicenter adult cohort” (31), ranked at the eighth position with 65 citations. This real-life study revealed high frequency (107/220, 48.6%) of non-infectious ophthalmologic AEs. Non-infectious conjunctivitis was the most common ocular AE, followed by ocular pruritus, blepharitis, xerophthalmia, and keratitis. Ocular AEs are well tolerated in most of patients, and only a small proportion of patients (10/238, 4.2%) discontinued the treatment of dupilumab at 6-month follow-up endpoint. A long-term real-life evidence entitled “A 48-week update of a multicentre real-life experience of dupilumab in adult patients with moderate-to-severe atopic dermatitis” (32), ocular AEs related to dupilumab were well tolerated with frequent remission in most cases.

Information about co-citation analysis were exhibited in Table 2, Figure 7B. Reference co-citation links represent the connection between two references that are both cited by the same article. The top 2 articles were the same as those in citation analysis, and had the highest co-citation times and total link strength, indicating a strong connection and close relatedness with other articles. This might be because the top-tier journal *New England Journal of Medicine* and *Lancet* preferred frontier research and innovative articles, and in return, these journals would bring a more influential and broader readership to the articles.

There were several limitations of this study. First, the data source was limited to the Web of Science Core Collection database, and only documents published in English were extracted, which may result in the inclusion bias. Moreover, to recognize contributions accurately, only original articles, case letters, and research letters were included in this study. Second, newly published articles have less time of exposure to allow for citations, which may lead to the underestimation of potential significant articles.

In summary, the present study presented a comprehensive bibliometric analysis concerning ocular AEs during the treatment of AD patients with dupilumab. The first study was published in 2016 by Oregon Health and Science University from the United States; the majority of publications were published in the past 3 years; *British Journal of Dermatology* published the highest number of articles; the United States was the country with the most publications; Sanofi (France) and Regeneron Pharmaceuticals (USA) were the leading organizations with most contributions; conjunctivitis was the most common ocular AE; the management of AD will continue to be the research hotspot and development trend in this area; the milestone research is the first article “Two Phase 3 Trials of Dupilumab vs. Placebo in Atopic Dermatitis” published in *New England Journal of Medicine*; most of the top 10 papers were mainly randomized, placebo-controlled phase 2 and phase 3 clinical trials and real-life large cohort studies.

Our results predicted that research on ocular AEs was the frontiers and promising field, and the management of AD was the great concern and difficulty and would be the future research direction. In conclusion, this study may help better understand ocular AEs in the dupilumab treatment of AD, and grasp the research trends and most influential topics in this field.

## Data Availability Statement

The raw data supporting the conclusions of this article will be made available by the authors, without undue reservation.

## Author Contributions

QNJ, JQ, and YPZ contributed to concept, design, literature search, and edited the manuscript. QNJ and KF contributed to data acquisition and analysis and statistical analysis. QNJ drafted the manuscript. QNJ, JQ, KF, and YPZ reviewed the manuscript. All authors contributed to the article and approved the submitted version.

## Conflict of Interest

The authors declare that the research was conducted in the absence of any commercial or financial relationships that could be construed as a potential conflict of interest.

## Publisher's Note

All claims expressed in this article are solely those of the authors and do not necessarily represent those of their affiliated organizations, or those of the publisher, the editors and the reviewers. Any product that may be evaluated in this article, or claim that may be made by its manufacturer, is not guaranteed or endorsed by the publisher.

## References

[1] Langan SM Irvine AD Weidinger S. Atopic dermatitis. Lancet. (2020) 396:345–60. 10.1016/S0140-6736(20)31286-1 32738956

[2] Laughter MR Maymone MBC Mashayekhi S Arents BWM Karimkhani C Langan SM . The global burden of atopic dermatitis: lessons from the global burden of disease study 1990-2017. Br J Dermatol. (2021) 184:304–9. 10.1111/bjd.19580 33006135

[3] Odhiambo JA Williams HC Clayton TO Robertson CF Asher MI ISAAC Phase Three Study Group. Global variations in prevalence of eczema symptoms in children from ISAAC phase three. J Allergy Clin Immunol. (2009) 124:1251–8. 10.1016/j.jaci.2009.10.009 20004783

[4] Weidinger S Beck LA Bieber T Kabashima K Irvine AD. Atopic dermatitis. Nat Rev Dis Primers. (2018) 4:1. 10.1038/s41572-018-0001-z 29930242

[5] Eckert L Gupta S Amand C Gadkari A Mahajan P Gelfand JM. The burden of atopic dermatitis in US adults: health care resource utilization data from the 2013 national health and wellness survey. J Am Acad Dermatol. (2018) 78:54–61. 10.1016/j.jaad.2017.08.002 29017738

[6] Silverberg JI Gelfand JM Margolis DJ Boguniewicz M Fonacier L Grayson MH . Patient burden and quality of life in atopic dermatitis in US adults: a population-based cross-sectional study. Ann Allergy Asthma Immunol. (2018) 121:340–7. 10.1016/j.anai.2018.07.006 30025911

[7] Leung DY. New insights into atopic dermatitis: role of skin barrier and immune dysregulation. Allergol Int. (2013) 62:151–61. 10.2332/allergolint.13-RAI-0564 23712284 PMC8609663

[8] Paton DM. Dupilumab: human monoclonal antibody against IL-4Rα for moderate to severe atopic dermatitis. Drugs Today. (2017) 53:477–87. 10.1358/dot.2017.53.9.2693150 29238761

[9] Thaçi D Simpson EL Beck LA Bieber T Blauvelt A Papp K . Efficacy and safety of dupilumab in adults with moderate-to-severe atopic dermatitis inadequately controlled by topical treatments: a randomised, placebo-controlled, dose-ranging phase 2b trial. Lancet. (2016) 387:40–52. 10.1016/S0140-6736(15)00388-8 26454361

[10] D'Erme AM Romanelli M Chiricozzi A. Spotlight on dupilumab in the treatment of atopic dermatitis: design, development, and potential place in therapy. Drug Des Devel Ther. (2017) 11:1473–80. 10.2147/DDDT.S113192 28553077 PMC5439982

[11] Beck LA Thaçi D Hamilton JD Graham NM Bieber T Rocklin R . Dupilumab treatment in adults with moderate-to-severe atopic dermatitis. N Engl J Med. (2014) 371:130–9. 10.1056/NEJMoa1314768 25006719

[12] Simpson EL Bieber T Guttman-Yassky E Beck LA Blauvelt A Cork MJ . Two phase 3 trials of dupilumab versus placebo in atopic dermatitis. N Engl J Med. (2016) 375:2335–48. 10.1056/NEJMoa1610020 27690741

[13] Wang Y Jorizzo JL. Retrospective analysis of adverse events with dupilumab reported to the United States food and drug administration. J Am Acad Dermatol. (2021) 84:1010–4. 10.1016/j.jaad.2020.11.042 33725800

[14] Ou Z Chen C Chen A Yang Y Zhou W. Adverse events of Dupilumab in adults with moderate-to-severe atopic dermatitis: a meta-analysis. Int Immunopharmacol. (2018) 54:303–10. 10.1016/j.intimp.2017.11.031 29182975

[15] Ludriksone L Elsner P Schliemann S. Acquired hypersensitivity to dupilumab: first case report. J Eur Acad Dermatol Venereol. (2019) 33:e482–3. 10.1111/jdv.15807 31310358

[16] Brumfiel CM Patel MH Zirwas MJ. Development of psoriasis during treatment with dupilumab: a systematic review. J Am Acad Dermatol. (2021). S0190-9622:00995-6. 10.1016/j.jaad.2021.05.013 34022319

[17] Zhu GA Kang KJW Chen JK Novoa RA Brown RA Chiou AS . Inflammatory alopecia in patients on dupilumab: a retrospective cohort study at an academic institution. J Eur Acad Dermatol Venereol. (2020) 34:e159–61. 10.1111/jdv.16094 31737955

[18] Chrétien B Dolladille C Alexandre J Fedrizzi S Lelong-Boulouard V Lambert JC . Dupilumab-associated arthralgia: an observational retrospective study in VigiBase. Br J Dermatol. (2021) 185:464–5. 10.1111/bjd.20138 33829495

[19] Akinlade B Guttman-Yassky E de Bruin-Weller M Simpson EL Blauvelt A Cork MJ . Conjunctivitis in dupilumab clinical trials. Br J Dermatol. (2019) 181:459–73. 10.1111/bjd.17869 30851191 PMC6850316

[20] Blauvelt A de Bruin-Weller M Gooderham M Cather JC Weisman J Pariser D . Long-term management of moderate-to-severe atopic dermatitis with dupilumab and concomitant topical corticosteroids (LIBERTY AD CHRONOS): a 1-year, randomised, double-blinded, placebo-controlled, phase 3 trial. Lancet. (2017) 389:2287–303. 10.1016/S0140-6736(17)31191-1 28478972

[21] de Bruin-Weller M Thaçi D Smith CH Reich K Cork MJ Radin A . Dupilumab with concomitant topical corticosteroid treatment in adults with atopic dermatitis with an inadequate response or intolerance to ciclosporin A or when this treatment is medically inadvisable: a placebo-controlled, randomized phase III clinical trial (LIBERTY AD CAFÉ). Br J Dermatol. (2018) 178:1083–101. 10.1111/bjd.16156 29193016

[22] Zirwas MJ Wulff K Beckman K. Lifitegrast add-on treatment for dupilumab-induced ocular surface disease (DIOSD): a novel case report. JAAD Case Rep. (2018) 5:34–6. 10.1016/j.jdcr.2018.10.016 30555883 PMC6280680

[23] Maymone MBC Laughter M Vashi NA Jones JD Hugh J Dunnick CA . The most cited articles and authors in dermatology: a bibliometric analysis of 1974-2019. J Am Acad Dermatol. (2020) 83:201–5. 10.1016/j.jaad.2019.06.1308 31279031

[24] Bickers DR Modlin RL. A review of the journal of investigative dermatology's most cited publications over the past 25 years and the use of developing bibliometric methodologies to assess journal quality. J Invest Dermatol. (2012) 132:1050–60. 10.1038/jid.2011.391 22330274 PMC4430846

[25] Zhang H Wang Y Zheng Q Tang K Fang R Wang Y . Research interest and public interest in melanoma: a bibliometric and google trends analysis. Front Oncol. (2021) 11:629–87. 10.3389/fonc.2021.629687 33680968 PMC7930473

[26] Sernicola A Gattazzo I Di Staso F Giordano D Capalbo A Persechino F . Treatment of refractory conjunctivitis associated to dupilumab with topical pimecrolimus applied to the eyelid skin. Dermatol Ther. (2019) 32:e13134. 10.1111/dth.13134 31639238

[27] Thyssen JP Toft PB Halling-Overgaard AS Gislason GH Skov L Egeberg A . Incidence, prevalence, and risk of selected ocular disease in adults with atopic dermatitis. J Am Acad Dermatol. (2017) 77:280–6. 10.1016/j.jaad.2017.03.003 28601391

[28] Tukler Henriksson J Coursey TG Corry DB De Paiva CS Pflugfelder SC. IL-13 stimulates proliferation and expression of mucin and immunomodulatory genes in cultured conjunctival goblet cells. Invest Ophthalmol Vis Sci. (2015) 56:4186–97. 10.1167/iovs.14-15496 26132778 PMC4495812

[29] Uchida H Kamata M Nagata M Fukaya S Hayashi K Fukuyasu A . Conjunctivitis in patients with atopic dermatitis treated with dupilumab is associated with higher baseline serum levels of IgE and TARC but not clinical severity in a real-world setting. J Am Acad Dermatol. (2020) 82:1247–9. 10.1016/j.jaad.2019.12.039 31884090

[30] Wollenberg A Ariens L Thurau S van Luijk C Seegräber M de Bruin-Weller M. Conjunctivitis occurring in atopic dermatitis patients treated with dupilumab-clinical characteristics and treatment. J Allergy Clin Immunol Pract. (2018) 6:1778–80. 10.1016/j.jaip.2018.01.034 29432961

[31] Faiz S Giovannelli J Podevin C Jachiet M Bouaziz JD Reguiai Z . Effectiveness and safety of dupilumab for the treatment of atopic dermatitis in a real-life French multicenter adult cohort. J Am Acad Dermatol. (2019) 81:143–51. 10.1016/j.jaad.2019.02.053 30825533

[32] Fargnoli MC Esposito M Ferrucci S Girolomoni G Offidani A Patrizi A . A 48-week update of a multicentre real-life experience of dupilumab in adult patients with moderate-to-severe atopic dermatitis. J Dermatolog Treat. (2020). 10.1080/09546634.2020.1773379. [Epub ahead of print]. 32436765

